# Notch regulates the angiogenic response via induction of VEGFR-1

**DOI:** 10.1186/2040-2384-2-3

**Published:** 2010-01-26

**Authors:** Yasuhiro Funahashi, Carrie J Shawber, Marina Vorontchikhina, Anshula Sharma, Hasina H Outtz, Jan Kitajewski

**Affiliations:** 1 Pathology, Obstetrics and Gynecology, and Herbert Irving Comprehensive Cancer Center, Columbia University Medical Center, New York, NY 10032, USA; 2 Tsukuba Research Laboratories, Eisai Co, Ltd, Ibaraki, Japan

## Abstract

Notch is a critical regulator of angiogenesis and arterial specification. We show that ectopic expression of activated Notch1 induces endothelial morphogenesis in human umbilical vein endothelial cells (HUVEC) in a VEGFR-1-dependent manner. Notch1-mediated upregulation of VEGFR-1 in HUVEC increased their responsiveness to the VEGFR-1 specific ligand, Placental Growth Factor (PlGF). In mice and human endothelial cells, inhibition of Notch signaling resulted in decreased VEGFR-1 expression during VEGF-A-induced neovascularization. In summary, we show that Notch1 plays a role in endothelial cells by regulating VEGFR-1, a function that may be important for physiological and pathological angiogenesis.

## Introduction

Vascular endothelial growth factor-A (VEGF-A) is essential to the multistep process of vascular development, and proper vessel formation in a variety of settings is exquisitely sensitive to levels of VEGF-A [1-4]. VEGF-A signals through two receptor tyrosine kinases: VEGFR-1 (flt1) and VEGFR-2 (flk1), while placenta growth factor (PlGF) signals exclusively through VEGFR-1. Both VEGF-A and PlGF induce endothelial cell proliferation, survival, and migration [3,5,6]. The role of VEGFR-1 in angiogenesis has largely been defined in terms of its opposition to VEGFR-2. VEGFR-2 is considered the primary VEGF-A receptor that drives angiogenesis, while VEGFR-1 has high binding affinity for VEGF-A but weak kinase activity. Thus, VEGFR-1 is thought to function mainly as a decoy receptor that sequesters VEGF-A [7-11]. This concept is supported by analysis of mouse models where deletion of *flt1 *led to vessel overgrowth and disruption of vascular patterning [12]. In addition, mice expressing a mutant allele of *flt1 *that lacks the tyrosine kinase domain (*flt1*^*TK*-/-^) did not exhibit the vascular patterning defects seen in *flt1*^-/-^mice, suggesting that in embryonic development, the kinase activity of VEGFR-1 was dispensable and that its predominant function is via its high affinity binding to VEGF-A [9]. Despite this, a positive function for VEGFR-1 in angiogenesis has been demonstrated in a variety of settings. *flt1*^*TK*-/- ^mice displayed defects in tumor vessel formation and metastasis [13,14], and inhibition of VEGFR-1 led to defects in neovascularization of the eye [15]. The signaling pathways that regulate VEGFR-1 expression in endothelial cells remain unclear.

Notch, a receptor that functions in cell fate decisions, has been shown to be downstream of VEGF-A in endothelial sprouting [16,17] and arterial specification [18,19]. The Notch proteins are highly conserved trans-membrane receptors that are required for normal embryonic development. In mammals, there are four Notch proteins (Notch1-4) that, upon binding with one of five ligands, termed Delta-like (Dll) and Jagged, are subject to a series of proteolytic cleavages by ADAM metalloproteases and gamma-secretase. Cleavage releases the intracellular domain of the Notch receptor, which translocates to the nucleus and functions as a transcriptional activator in complex with the transcription factors CSL (CBF1, Su(H), Lag-2), Mastermind, and histone acetyltransferases. To date, the importance of the Notch pathway in regulating endothelial cell response to VEGF-A has been studied with respect to its effect on VEGFR-2, as it has been shown that Delta-like 4 (Dll4) signaling represses VEGFR-2 expression [16,20,21]. Current models assert a role for Dll4 in restricting sprouting angiogenesis [20,22-24], but have not identified the Notch receptors that are important for this effect, or whether Notch signaling can function positively in endothelial cell morphogenesis. In addition, whether Notch signaling through a particular receptor can regulate VEGFR-1 expression in endothelial cells has not been defined.

Using ectopic expression as well as protein-based, and pharmacological loss of Notch function, we show that VEGFR-1 expression is downstream of Notch signaling in endothelial cells. Furthermore, we define a positive role for Notch signaling in VEGF-driven morphogenesis of endothelial cells via promotion of cell extension which we demonstrate requires upregulation of VEGFR-1. Coincident with the Notch-mediated upregulation of VEGFR-1, we report Notch signaling enhances endothelial cell responsiveness to PlGF. Finally, in an assay of VEGF-A induced dermal angiogenesis, we show that a protein based Notch inhibitor, the Notch1 decoy, can reduce VEGFR-1 levels in neovessels. Collectively, our data define a role for Notch in mediating the response of endothelial to angiogenic stimuli by regulation of VEGFR-1.

## Materials and methods

### Reagents, Expression Vectors

ZD1893, PD166866, and SU5416 are from Eisai Co., Ltd. Compound E was obtained from the Korean Research Institute of Chemical Technology. PlGF was obtained from Research Diagnostics Institute. N1IC [25], LacZ, and VEGF-A constructs were engineered into pAdlox vector and adenovirus stocks were produced [26]. Notch1 decoy has been described [27]. Briefly, the extracellular domain of rat Notch1 (bp 241-4229, accession no. X57405) was fused to human IgG Fc and engineered into pAdlox vector (Ad-Notch1 decoy) and adenovirus stocks generated.

### Cell Culture, Adenoviral Infections, retroviral infections, siRNA

HUVEC were isolated from human umbilical vein as described [28] and cultured in complete medium (EGM-2 Bullet kit, LONZA) on porcine type I collagen (Nitta Gelatine). KP1/VEGF^121 ^cells were provided by Eisai Co., Ltd, [27] and maintained in RPMI 1640 containing 10% FBS. HUVEC were infected with Ad-LacZ, Ad-N1IC, Ad-VEGF-A, Ad-GFP, or Ad-Notch1 decoy at a MOI of 40. HUVEC were co-infected with Ad-LacZ and Ad-Notch1 decoy at a MOI of 40 for each virus. HUVEC infected with Ad-LacZ at a MOI of 80 served as a control. Retroviral control and N1IC-expressing HUVEC lines were generated as previously described [29]. Control, VEGFR-1, and VEGFR-2 siRNA (Santa Cruz) were introduced into HUVEC using Effectene Reagent (Qiagen). Total RNA or cell lysate was harvested 48 hours after siRNA transfection.

### RT-PCR

HUVEC were seeded on type I collagen gels two days after adenoviral infection or retroviral infection and 5 days later total RNA was isolated with RNeasy mini kit (Qiagen). First-strand cDNA was synthesized using SuperScript First-Strand Synthesis System (Invitrogen). For RT-PCR, primers were designed to recognize human and mouse transcripts of VEGFR-1, VEGFR-2, VEGF-A, PlGF, GAPDH and beta-actin, (primer sequence available upon request). PCR used Platinum Taq DNA polymerase (Invitrogen) and reactions performed for 25 or 30 cycles. Reactions were performed in triplicate.

### Western Blotting

HUVEC were cultured on type I collagen gels for 5 days in complete medium, then starved in serum free medium for 48 hours and cell lysates were collected with TENT lysis buffer. Western blots were performed using antibodies against Flt1 (C-17, Santa Cruz), Flk1 (C-1158, Santa Cruz), and alpha-tubulin (Sigma). To validate Notch1 decoy secretion, serum-free medium from adenovirally transduced HUVEC was used for western blot analysis using an antibody against the Fc tag (Pierce).

### HUVEC Morphogenesis Assay

Adenovirus infections were performed two days before seeding on porcine type I collagen, and HUVEC morphogenesis was assessed by microscopy after 5 days, as described [30]. Extensions were scored as number of cells with single or multiple processes per 10× microscopy field. Processes were defined as extensions at acute angles to the cell body that alter normal HUVEC morphology. For each experiment, at least five 10× fields of cultures from each condition were scored. Kinase inhibitors were added to the medium one hour after HUVEC seeding, and PlGF was added at the time of HUVEC seeding. For knockdown experiments, siRNA was transfected two days after adenvoviral infection and the cells were cultured for three days before assessment of HUVEC with cellular extensions. Cell number was measured using Cell Counting Kit-8 (Dojindo).

### Mouse DAS Assay

The Dorsal Air Sac (DAS) assay was performed as described [31]. Millipore chambers were packed with 5.0 × 10^6 ^KP1/VEGF^121 ^cells that were transduced (60 MOI) with either Ad-GFP or Ad-Notch1 decoy and transplanted into a DAS of C57BL/6 mice. Mice were sacrificed four days after implantation and implants harvested and embedded in OCT. Each group consisted of at 3-5 mice, and experiments done in triplicate.

### Immunohistochemistry

5-μm serial sections of KP1/VEGF^121 ^implants were immunostained as described [32]. The following antibodies were used: PECAM (553370, BD Pharmingen), Flt1 (AF417, R&D Systems), Flk1 (AF644, R&D Systems). Quantitative analysis of CD31, Flk1, and Flt1 immunostaining of skin was performed on serial sections using an Eclipse E800 microscope and Nikon DXM 1200 camera, with ImagePro Plus software (Silver Spring, MD). Measurements were made in five different areas in each sample at 20× magnification and average density ratio was determined by dividing the area of specific staining by the total area of the smooth muscle layer.

### Flow Cytometry

2 × 10^5 ^HUVEC were seeded per well in a collagen-coated 6-well plate. 24 hrs after seeding, cells were stimulated with 50 ng/ml recombinant VEGF-A (R&D Systems) in complete medium, with or without 200 nM Compound E (Korean Research Institute). DMSO was used to treat control cells. 24 hours post-stimulation, cells were harvested with cold PBS, washed, and incubated with rabbit-anti VEGFR-1 (Santa Cruz) for 45 minutes at 4°C. After washing, cells were labeled with anti-rabbit-APC (Jackson Immunoresearch) for 25 minutes at 4°C. Flow cytometry was performed and 10,000 cells per experimental group were counted using FACSCalibur and CellQuestPro acquisition software (BD Biosciences).

### Statistical Analysis

Data were expressed as mean plus or minus SEM. Statistical analysis was performed by 2-tailed student *t *test. P value of less than 0.05 is indicated with ⋆, P value of less than 0.02 is indicated with *. All data shown is representative of at least 3 independent experiments.

## Results

### Notch signaling induced cellular extensions and VEGFR-1 expression in HUVEC

**Figure 1:**
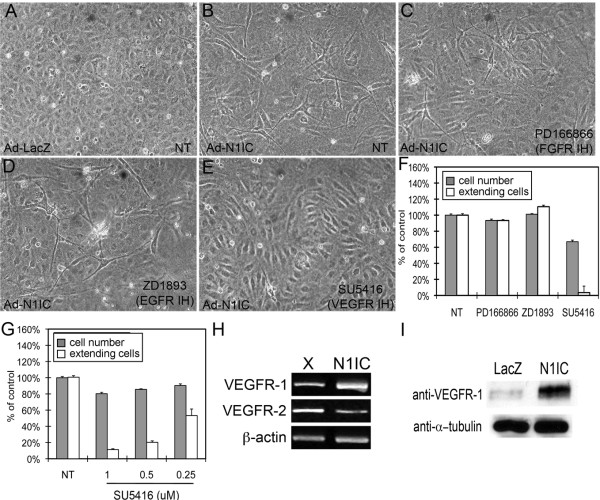
**Notch signaling induced cellular extensions and VEGFR-1 expression in HUVEC**. HUVEC were transduced with either Ad-LacZ or Ad-N1IC (MOI 40) and seeded on collagen type I gel for five days. Representative images from each cell culture are shown (10× magnification). (A) Ad-LacZ HUVEC did not form extensions, (B) while Ad-N1IC HUVEC underwent morphological changes as seen by the sprouting of extensions into the underlying matrix. (C) Morphological differentiation of Ad-N1IC HUVEC was not affected by the addition of 1 μM PD166866 (FGFR inhibitor), or (D) 1 μM ZD1893 (EGFR inhibitor). (E) The addition of 0.5 μM SU5416 (a VEGFR inhibitor), suppressed the morphological differentiation of Ad-N1IC HUVEC. (F) Quantification of the effect of the different tyrosine kinase inhibitors on Notch-induced cellular extensions and cell number. (G) Quantification of the effect of increasing amounts of the VEGFR inhibitor (SU5416) on Notch-induced cellular extensions and cell number. For morphogenesis assays, HUVEC with sprouting extensions per 10× microscopy field were counted, for five separate fields. Data is representative of the mean plus or minus SEM of three separate experiments, relative to control. Cell number was determined as percent of control using colorimetric cell proliferation kit. (H) RT-PCR of RNA isolated from mock (X) or N1IC-expressing retrovirally transduced HUVEC lines for VEGFR-1 (30 cycles), VEGFR-2 (30 cycles) and β-actin (25 cycles) (I) Western blot of total cell lysate from Ad-LacZ or Ad-N1IC transduced HUVEC to detect VEGFR-1 protein expression. Detection of α-tubulin was used as a loading control.

We investigated whether Notch1 signaling could affect endothelial cell morphogenesis, as manifested by the appearance of VEGF-A- or Notch-induced cellular extensions from human umbilical vein endothelial cells (HUVEC)[27,30]. HUVEC were transduced with an adenovirus expressing the intracellular domain of Notch1 (Ad-N1IC) or a control plasmid (Ad-LacZ), and seeded on three-dimensional Type I collagen gels. The N1IC construct encodes a constitutively active, gamma-secretase cleavage-independent form of Notch1 [25]. Ad-infected HUVEC were evaluated and scored for the number of cells forming cellular extensions per field, as well as for cell number, three days after seeding. We found that Ad-N1IC HUVEC displayed an increase in cellular extensions compared to Ad-LacZ cells (Figure 1A-B). Because HUVEC were cultured in the presence of multiple growth factors, we determined if this effect was due to signaling through a particular receptor using specific small molecule inhibitors for FGFR, EGFR and VEGFR. While inhibitors to fibroblast growth factor receptor (FGFR) or epidermal growth factor receptor (EGFR) did not inhibit extensions in Ad-N1IC HUVEC, SU5416, an inhibitor of VEGFR-1 and VEGFR-2, suppressed N1IC-induced extensions (Figure 1C-E). The reduction in extensions seen with VEGFR inhibition was accompanied by a 33% decrease in cell number (Figure 1F). However, the extension defect seen in cells treated with SU5416 was more dramatic than the decrease in cell number. Inhibition of N1IC-induced extensions in HUVEC with SU5416 was dose-dependent (Figure 1G). In endothelial cells, Notch signaling is known to down-regulate VEGFR-2 expression, thus we hypothesized that the Notch-induced extensions were mediated by VEGFR-1 [20,29]. Consistent with this hypothesis, we found that N1IC expression induced expression of VEGFR-1 transcripts (Figure 1H) and protein (Figure 1I). We also observed that N1IC suppressed VEGFR-2 transcripts (Figure 1H), similar to previous publications [20,24]. These data suggest that induction of extensions in Ad-N1IC HUVEC is dependent on VEGFR-1, not VEGFR-2.

### Notch1-induced extensions in HUVEC is enhanced by PlGF

**Figure 2:**
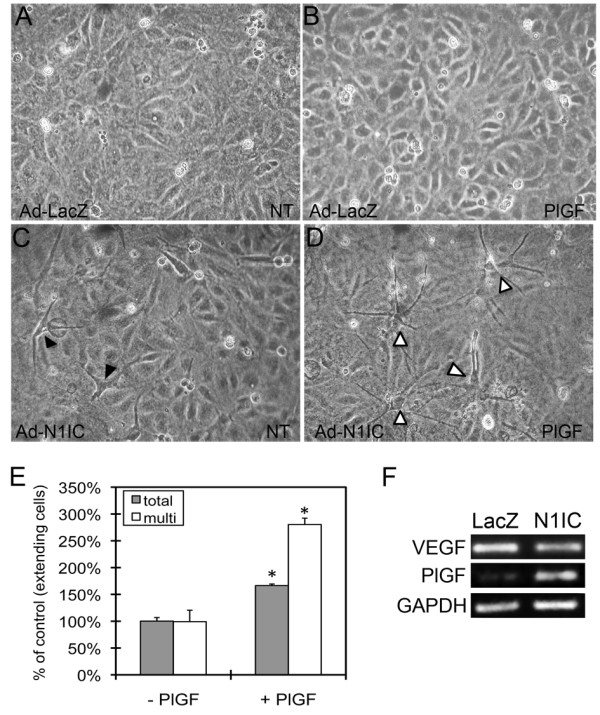
**Notch1-induced HUVEC morphological changes were enhanced by the VEGFR-1-specific ligand PlGF**. HUVEC were transduced with Ad-LacZ or Ad-N1IC (MOI 40) and cultured on collagen type I gels in serum free medium with or without 50 ng/ml PlGF and evaluated for the formation of cellular extensions. Representative images from each cell culture are shown (10× magnification). (A-B) Ad-LacZ HUVEC remained a homogenous monolayer in the absence (NT) or presence of PlGF. (C) In the absence of PlGF, Ad-N1IC HUVEC undergo morphological differentiation characterized by one or two extensions per cell (black arrowheads). (D) In presence of PlGF, there is an increase number of Ad-N1IC HUVEC undergoing morphological changes and the number of extensions per cell (open arrowheads). (E) Quantification of total number of Ad-N1IC HUVEC with cellular extensions and cells with multiple (>3) processes with or without PlGF. Number of cells with cellular extensions were counted per 10× field, for five separate fields. Data is representative of the mean plus or minus SD of three separate experiments. * P < 0.01 compared with cultures without PlGF. (F) RT-PCR of RNA isolated from Ad-LacZ and Ad-N1IC HUVEC for VEGF-A (22 cycles), PlGF (25 cycles) and GAPDH (22 cycles), as a control.

Because ectopic expression of Notch1 induced VEGFR-1, we hypothesized that these cells would exhibit increased responsiveness to the PlGF. Ad-N1IC or control Ad-LacZ HUVEC were cultured on Type I collagen gels in serum free medium, with or without 50 ng/ml PlGF. While PlGF did not induce extensions in control cells (Figure 2A-B), addition of PlGF to N1IC-expressing HUVEC enhanced extensions (Figure 2C-D). Extensions in N1IC-expressing HUVEC were generally one or two processes from a single cell (Figure 2C, black arrowheads), while addition of PlGF led to a near threefold increase in cells with more than two extensions (Figure 2D, open arrowheads, Figure 2E). Notch was found to increase the levels of PlGF, but not VEGF-A transcripts in HUVEC (Figure 2F), which may contribute to the extensions induced in HUVEC expressing N1IC in the absence of exogenous PlGF. Thus, while PlGF alone is not sufficient to induce HUVEC morphogenesis, in the context of activated Notch1 signaling, PlGF can enhance extensions in these cells, likely due to increased expression of VEGFR-1.

### Reduced VEGFR-1, but not VEGFR-2, inhibited Notch-induced extensions in HUVEC

**Figure 3:**
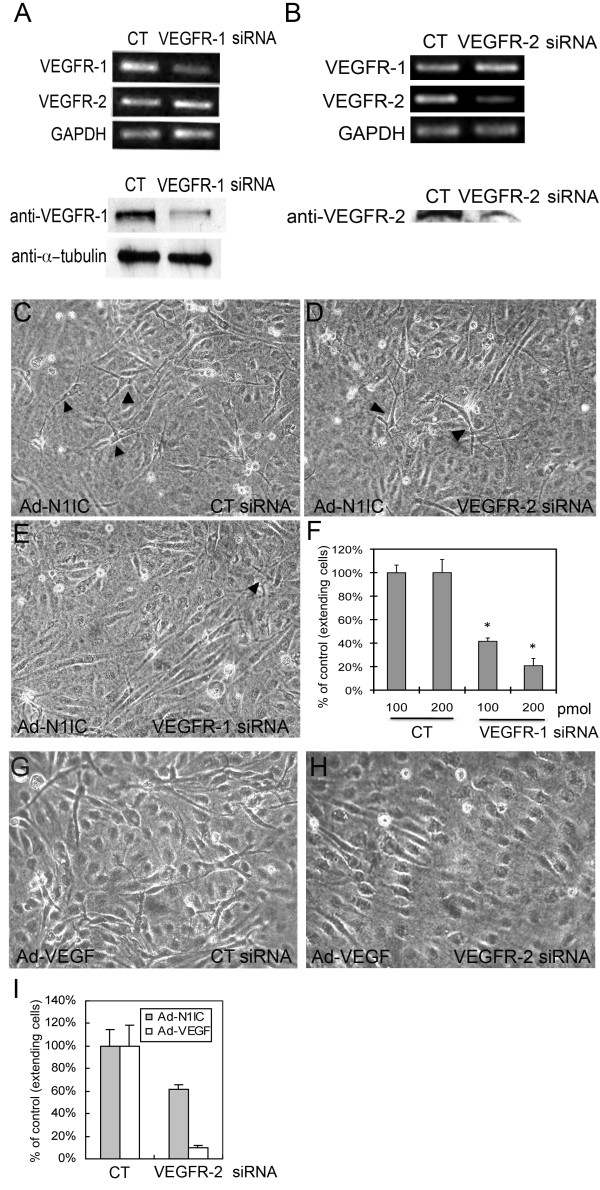
**Notch-induced sprouting in HUVEC did not depend on VEGFR-2 expression**. (A, B) Ad-N1IC HUVEC were transfected with 200 pmol of control, VEGFR-1 or VEGFR-2 siRNA and cultured on collagen gels. (A, upper panels) RT-PCR of Ad-N1IC HUVEC transfected with control (CT), or VEGFR-1 siRNA for VEGFR-1 and VEGFR-2 (25 cycles each) and (lower panels) western blot with an antibody against VEGFR-1 demonstrated decreased transcript and protein levels of VEGFR-1 in cells transfected with VEGFR-1 siRNA relative. VEGFR-2 transcripts were unchanged by VEGFR-1 siRNA. GAPDH (25 cycles) and α-tubulin were used as controls for the RT-PCR and western blot, respectively. (B, upper panels) RT-PCR of Ad-N1IC HUVEC transfected with control (CT) or VEGFR-2 siRNA for VEGFR-1, VEGFR-2 and GAPDH (25 cycles each) and (lower panels) western blot with an antibody against VEGFR-2. VEGFR-2 siRNA suppressed VEGFR-2 transcripts and protein, but did not alter VEGFR-1 transcripts. (C-E) Three days after transfection with control, VEGFR-1 or VEGFR-2 siRNAs, Ad-N1IC HUVEC were evaluated for morphological changes (compare cell extensions, black arrowheads). Representative images are shown (10× magnification). (C) N1IC-induced cellular extensions was unaffected by control siRNA. (D) VEGFR-2 siRNA resulted in a modest decrease in Notch-induced morphological changes. (E) VEGFR-1 siRNA suppressed the morphological differentiation of Ad-N1IC HUVEC. (F) Quantification of the effect of either 100 or 200 pmol VEGFR-1 siRNA on Notch-induced HUVEC undergoing morphological changes. (G, H) Ad-VEGF-A (VEGF) HUVEC were transfected with 200 pmol of control (CT) or VEGFR-2 siRNA and cultured on collagen gels for three days. (G) Ad-VEGF HUVEC underwent morphological changes with control siRNA. (H) VEGFR-2 siRNA suppressed Ad-VEGF HUVEC morphological changes. (I) Quantification of the effect of 200 pmol VEGFR-2 siRNA on VEGF-A or Notch-induced morphological changes. VEGFR-2 siRNA only modestly affected Ad-N1IC HUVEC morphogenesis, while it strongly suppressed Ad-VEGF HUVEC morphogenesis. (F, I) Cells with extensions were counted per 10× field, for five separate fields. Data is representative of the mean plus or minus SD of three separate experiments. * P < 0.01 compared with control.

Though VEGFR-2 expression in endothelial cells is downregulated by Notch signaling (Figure 1H), the possibility that cellular extensions in N1IC-expressing HUVEC is the result of residual VEGFR-2 and that enhanced extensions with PlGF is due to intermolecular crosstalk between VEGFR-1 and VEGFR-2 [33,34], could not be excluded. To examine these possibilities, Ad-N1IC HUVEC were transfected with VEGFR-1, VEGFR-2, or a control (CT) siRNA and cultured on collagen gels to determine the effect of decreased expression of individual receptors on Notch-induced extensions. Compared to Ad-N1IC HUVEC treated with control siRNA, transcript and protein levels of VEGFR-1 in cells transfected with VEGFR-1 siRNA were reduced, as shown by RT-PCR and western blot (Figure 3A). VEGFR-2 expression was unaltered by the VEGFR-1 siRNA (Figure 3A, left). Similarly, VEGFR-2 siRNA was specific for VEGFR-2, resulting in decreased transcripts and protein expression, but VEGFR-2 siRNA did not affect levels of VEGFR-1 transcripts (Figure 3B). Transfection of control siRNA did not affect Notch-induced extensions (Figure 3C). VEGFR-2 siRNA resulted in only a modest decrease in the Notch-induced extensions compared to its ability to suppress VEGF-A-induced extensions (Figure 3C, D, G, H). However, transfection of VEGFR-1 siRNA significantly reduced Notch-induced extensions in HUVEC (Figure 3E). Quantification of extensions in these cultures demonstrated that a lower dose of VEGFR-1 siRNA resulted in a less dramatic decrease in the number of extensions compared to control than a higher dose of VEGFR-1 siRNA (42% vs 21% of control, respectively, Figure 3F). Because VEGFR-2 siRNA drastically reduced VEGF-A-induced extensions (Figure 3H, I), but has only a modest affect on Notch-induced extensions (Figure 3D, I), our results support the possibility that Notch1 acts downstream of VEGF-A/VEGFR-2 signaling and induces endothelial cell morphogenesis via VEGFR-1.

### Expression of VEGFR-1 in neovessels was decreased when Notch signaling is inhibited

**Figure 4:**
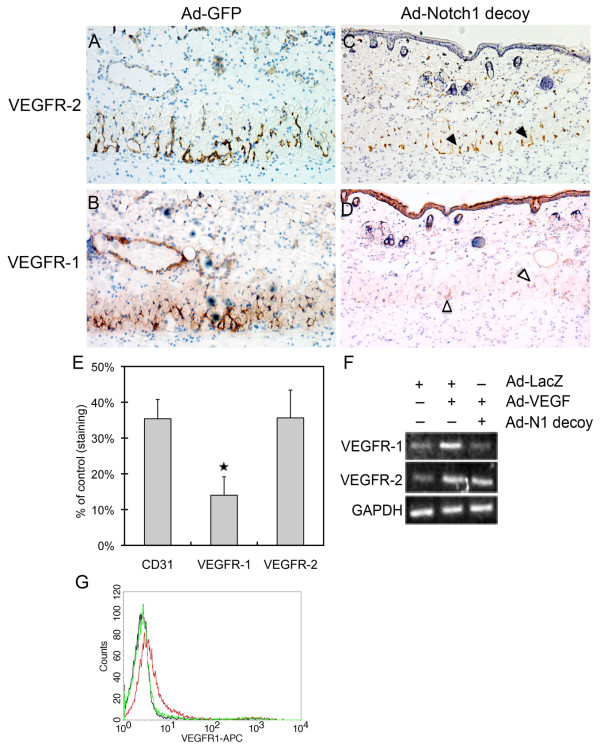
**Inhibition of Notch signaling decreased VEGFR-1 expression in neovessels**. Chambers packed with VEGF-expressing KP1 pancreatic tumor cells (KP1/VEGF-A^121^) transduced with either Ad-GFP or Ad-Notch1 decoy were implanted under the dorsal skin of wild-type C57BL/6 mice. Four days after implantation, chambers were removed for immunohistochemistry of the overlying skin. Representative pictures are shown (20× magnification). (A-B) VEGFR-1 and VEGFR-2 expression in the smooth muscle cell layer of the skin overlying implants expressing Ad-GFP. (C) Expression of VEGFR-2 was detected in implants expressing the Notch1 decoy despite decreased vessel density (black arrowheads). (D) Expression of VEGFR-1 in implants expressing the Notch1 decoy was significantly reduced in the smooth muscle cell layer (open arrowheads). (E) Reduced VEGFR-1 expression relative to CD31 and VEGFR-2 was observed in skin overlying implants expressing the Notch1 decoy compared to those expressing Ad-GFP. Quantitative analysis of immunostaining intensity for each antibody, relative to Ad-GFP implants (set at 100%), was determined in five different areas of each sample. The average density ratio was determined by dividing the area of staining by the total area of the smooth muscle layer. Each group (Ad-GFP versus Ad-Notch1 decoy) consisted of 3-5 mice, and experiments were done in triplicate. Data represents the mean staining intensity in Notch1 decoy implants, expressed as percent of control staining, plus or minus SD. ⋆ P < 0.05. (F) Notch1 decoy expression in HUVEC reduced VEGF-induced VEGFR-1 expression. Adenovirus transduction of VEGF-A in HUVEC increased the expression of VEGFR-1 and VEGFR-2 as determined by RT-PCR (25 cycles) compared to control (Ad-LacZ) cells. When Ad-VEGF-A (VEGF) HUVEC were co-transduced with the Notch1 decoy, transcript levels of VEGFR-1 were reduced, while VEGFR-2 was unaffected. (G) Gamma secretase inhibitor (GSI) reduces VEGF-induced surface expression of VEGFR-1 in HUVEC. Stimulation of HUVEC with 50 ng/ml VEGF-A induced surface expression of VEGFR-1 (red) compared to control cells (black). Co-incubation with GSI (Compound E) inhibited the VEGF-A-induction of VEGFR-1 (green). 10,000 cells per experimental group were examined by FACs.

The role of Notch in physiological angiogenesis was evaluated using a Dorsal Air Sac (DAS) assay, where a chamber containing VEGF^121^-expressing pancreatic KP1 tumor cells (KP1/VEGF^121^) is implanted under the dorsal skin of a mouse and the overlying dermis evaluated for ingrowth of vessels [31]. In this assay, we used a protein-based inhibitor of Notch signaling that encodes the extracellular EGF-like repeat domain of Notch1 fused to the human Fc domain, which we call the 'Notch1 decoy' [27]. We have shown that angiogenesis is induced in the smooth muscle layer of the skin overlying the KP1/VEGF^121 ^chamber, but is inhibited when KP1/VEGF^121 ^cells also express the Notch1 decoy via adenoviral transduction (Ad-Notch1 decoy) as compared to control (Ad-GFP) [27]. Thus, in this assay, VEGF^121^-induced angiogenesis was dependent on Notch signaling [27]. To evaluate endothelial VEGFR-1 and VEGFR-2 expression, cross sections of skin from the DAS assay were immunostained with antibodies against VEGFR-1, VEGFR-2 or CD31. Both VEGFR-1 and VEGFR-2 were expressed in the neovessels of control KP1/VEGF^121 ^implants transduced with Ad-GFP (Figure 4A-B). VEGFR-2 staining was detected in implants transduced with the Notch1 decoy, though its expression was decreased, reflecting a decrease in vessel density (Figure 4C, black arrowheads). However, expression of VEGFR-1 in Notch1 decoy-expressing implants was significantly reduced compared to control, and seen only faintly in the smooth muscle cell layer (Figure 4D, open arrowheads). To normalize for decreased vessel density in implants expressing the Notch1 decoy, the intensity of VEGFR-1 and VEGFR-2 signals was compared to that of the endothelial cell marker CD31 by quantitative analysis of immunohistochemical signal for each antibody (Figure 4E). While VEGFR-2 expression was decreased in the Notch1 decoy-expressing implant to the same extent as CD31 (36% and 35% of Ad-GFP implants, respectively), VEGFR-1 expression was decreased by a greater extent than either VEGFR-2 or CD31 (14% of control). This suggests that expression of the Notch1 decoy in KP1/VEGF^121 ^cells reduced vessel number, but not VEGFR-2 expression in endothelial cells, whereas the decrease in VEGFR-1 expression was independent of the decrease in vessel number. Thus, in VEGF-induced neovascularization, VEGFR-1 expression is dependent on Notch signaling.

This regulation was also found in cultured HUVEC, where VEGF-A-induced expression of VEGFR-1 was reduced by co-expression of the Notch1 decoy, as shown by RT-PCR (Figure 4F). In contrast, induction of VEGFR-2 by VEGF-A in HUVEC was unaffected by the Notch1 decoy (Figure 4F). Similarly, VEGFR-1 expression on the surface of VEGF-A-treated HUVEC was suppressed by treatment with a gamma secretase inhibitor (GSI), Compound E, as analyzed by flow cytometry (Figure 4G). Thus, two means of Notch inhibition were used to establish that VEGF-A induces Notch signaling which in turn regulates VEGFR-1 and that this regulatory pathway is active in both cultured endothelial cells and neovessels in mice.

## Discussion

Our results show that VEGFR-1 is downstream of Notch1 signaling in endothelial cells. We identify a positive role for Notch signaling in endothelial morphogenesis via the induction of cellular extensions mediated by VEGFR-1. Supporting this conclusion is the observation that Notch increases VEGFR-1 levels and this increase correlated with increased endothelial responsiveness to the VEGFR-1-specific ligand, PlGF. Using a protein-based Notch inhibitor, Notch1 decoy, or a gamma secretase inhibitor, we demonstrate that perturbation of endogenous Notch signaling resulted in reduced VEGFR-1 expression. Thus, loss- and gain- of function studies show that Notch signaling regulates VEGFR-1 expression in HUVEC and dermal neovessels.

Previous studies have demonstrated a role for the Notch ligand, Dll4, in inhibiting a tip cell phenotype in the developing vasculature of the retina [16,17]. In addition, Harrington et al [24] have shown that VEGFR-1 is upregulated by Dll4, and demonstrated that Dll4 signaling inhibited sprout length in a HUVEC tubulogenesis assay. The authors suggest that Dll4 signaling inhibits angiogenesis by inducing VEGFR-1 [24]. In summary, previous studies have found a negative role for Notch signaling in endothelial cell sprouting, and have focused on this signaling pathway at the level of the ligand, Dll4. However, in these studies, the Notch receptor responsible for these effects is not defined and the possibility of divergent effects of different Notch receptors is not addressed. By focusing on the effects of Notch signaling at the level of the receptor, our results add new insights to the role of Notch and VEGFR-1 in sprouting angiogenesis. In contrast to previous studies, our data suggest that in some settings, Notch signaling may play a positive role in endothelial cell extension of filopodia-like structures via its regulation of VEGFR-1 and supports a novel role Notch1-mediated regulation of VEGFR-1 in endothelial cell morphogenesis.

It has recently been found that VEGFR-1 promotes vascular sprout formation and branching morphogenesis [35,36]. Kearney et al [35] propose that this results from VEGFR-1 binding to VEGF-A, thereby regulating the amount of VEGF-A that is available to interact with VEGFR-2. They also show that soluble VEGFR-1 (sVEGFR-1) can promote sprout formation and migration. The positive effect of Notch signaling on HUVEC sprouting that we report may therefore be due to its effect on VEGFR-1, and subsequently, on local levels and availability of VEGF-A. This may particularly be the case if the predominant effect of Notch signaling is due to regulation of sVEGFR-1. In general, the relative proportion of the membrane bound and secreted isoform of VEGFR-1 does not change significantly (data not shown, and Kappas et al [36]), therefore, we cannot entirely exclude the possibility that Notch-induced sprouting in HUVEC is due to sequestration of VEGF-A. However, we show that Notch-induced sprouting in HUVEC is enhanced in the presence of PlGF, a VEGFR-1 specific ligand, suggesting that signaling through the VEGFR-1 receptor itself, and not simply its function as a 'VEGF-A sink,' may be responsible for Notch-mediated sprouting. This is further supported by the fact that VEGFR-1 siRNA inhibited Notch-induced sprouting in HUVEC while VEGFR-2 siRNA had only a modest effect. Thus, our data support the conclusion that activation of Notch signaling in HUVEC can induce extensions via VEGFR-1, and highlight the possibility that Notch signaling may act through VEGFR-1 to have a positive effect on endothelial cell morphogenesis.

It has been reported that inhibition of VEGFR-1 in the developing retina does not effect sprouting and filopodia extensions in endothelial cells [3,16]. In the retina, endothelial tip cell filopodia are guided by a gradient of VEGF-A provided by a template of astrocytes [3,37]. However, in our model of in vitro sprouting in HUVEC, as well as in many *in vivo *settings of physiological and pathological angiogenesis, the source of VEGF-A is likely to be more diffuse. Notch-mediated sprouting via regulation of VEGFR-1 may constitute a mechanism for endothelial cell morphogenesis that is important in settings where Notch1 is highly expressed in the vasculature and where expression of VEGF-A is more global, and endothelial cell sprouting less controlled, than in formation of the retinal plexus. In addition, our finding that Notch-induced sprouting in endothelial cells is enhanced by PlGF may be relevant in angiogenic settings where PlGF is a major angiogenic factor. Since PlGF is upregulated in pathological conditions by various stimuli [38-40], and contributes to the angiogenic switch in various pathologies [6,41,42], Notch-mediated upregulation of VEGFR-1 may prove an important step in disease progression in these contexts. Furthermore, our finding that blockade of Notch signaling using a protein-based inhibitor of Notch1 (Notch1 decoy) resulted in decreased expression of VEGFR-1 in an *in vivo *model of angiogenesis may have important implications for the efficacy of inhibition of Notch signaling in settings where VEGFR-1 expression is prominent, such as in certain tumor types and in the initiation of premetastatic niches [43-45].

## Competing interests

The authors declare that they have no competing interests.

## Authors' contributions

YF, CJS, MV, AS and HHO. performed experiments; HHO, CJS and YF, analyzed results and made the figures; HHO, YF and CJS wrote the manuscript; JK provided oversight for the research and guidance in preparation of the manuscript. All authors read and approved the final manuscript.
